# Analysis of intrinsic evolutionary factors leading to microendemic distributions in New Caledonian leaf beetles

**DOI:** 10.1038/s41598-023-34104-z

**Published:** 2023-04-27

**Authors:** Leonardo Platania, Jesús Gómez-Zurita

**Affiliations:** 1 Botanical Institute of Barcelona (CSIC-Ajuntament Barcelona), Pg. del Migdia S/N, 08038 Barcelona, Spain; 2 Universitat Pompeu Fabra, 08003 Barcelona, Spain

**Keywords:** Biogeography, Speciation

## Abstract

Microendemicity, or the condition of some species having local ranges, is a relatively common pattern in nature. However, the factors that lead to this pattern are still largely unknown. Most studies addressing this issue tend to focus on extrinsic factors associated with microendemic distributions, such as environmental conditions, hypothesising a posteriori about underlying potential speciation mechanisms, linked or not to these conditions. Here, we use a multi-faceted approach mostly focusing on intrinsic factors instead, namely diversification dynamics and speciation modes in two endemic sibling genera of leaf beetles with microendemic distributions, *Taophila* and *Tricholapita*, in a microendemicity hotspot, New Caledonia. Results suggest that the diversification rate in this lineage slowed down through most of the Neogene and consistently with a protracted speciation model possibly combined with several ecological and environmental factors potentially adding rate-slowing effects through time. In turn, species accumulated following successive allopatric speciation cycles, possibly powered by marked geological and climatic changes in the region in the last 25 million years, with daughter species ranges uncorrelated with the time of speciation. In this case, microendemicity seems to reflect a mature state for the system, rather than a temporary condition for recent species, as suggested for many microendemic organisms.

## Introduction

Microendemicity or narrow endemism is a relative concept that qualifies the global range of a particular species as small^1^. The literature on biodiversity includes many examples of microendemic species, species with narrow geographic distributions often recognisable owing to adaptations to their relatively small habitats. The interest in these species typically arises from conservation concerns, since they are perceived as more vulnerable than related species with larger ranges to threats associated with climate change^2^, genetic impoverishment^3^, outcompetition by invasive species^4^, or habitat disturbance^5^, to mention the most obvious. Many groups of organisms have examples of microendemic species, and some seem particularly prone to exhibit this pattern, including palms, frogs, millipedes, scorpions, geckos, and others^6–10^. However, what makes this biodiversity pattern particularly intriguing is that there are geographic regions that tend to concentrate these microendemisms, regions showing high rates of microendemicity across different groups of organisms. This pattern has been typically associated with special characteristics of the region, including age, isolation, topography or climate^11^. For example, the Espinhaço range in Brasil and the neighbouring Cerrado and Atlantic forests could be considered important plant microendemicity hotspots in South America^12–14^. The Cape Region and the Drakensberg range in South Africa are also known for plant microendemicity, but several groups of animals reflect this pattern too^15,16^. However, the best examples of areas characterised by generalised microendemicity across taxonomic groups are two tropical, ancient continental islands: Madagascar and New Caledonia^1,17^. The origin of endemic diversity in these two islands followed different paths—old and vicariant in the case of Madagascar^18^, recent and probably following transmarine colonisations in the case of New Caledonia^19^, but both island biotas seemingly underwent high diversification in situ^10,20–25^. Therefore, studies focusing on these areas have the potential to unveil general mechanisms responsible for geographic patterns of microendemicity.

Not much is known about the processes that underlie the pattern of regional microendemicity. For example^11^, proposed a mechanistic model entirely relying on predicted past climatic shifts and the topography and hydrology of Madagascar to explain species distribution patterns. A similar argument, combining climatic cycles and geographic factors, was also invoked to explain the accumulation of narrow endemics in the Mediterranean^26^. However, the simultaneity of geographic and taxonomic elements suggests that a more generalised answer may lie in the concourse of both extrinsic factors related to the physical and historical characteristics of the region where microendemicity is common, and intrinsic factors related to the mode of diversification of the groups that tend to exhibit microendemicity^27,28^.

Among extrinsic factors, two apparently opposing major theories are generally considered, one relating these patterns to geological and climatic habitat stability^14,29,30^, and one stressing changes in climate, orography and opportunities for dispersal as the engine for allopatric speciation^11,19,26,31^. However, these seemingly opposed theories can be reconciled in models that take scale into account, allowing for the persistence of lineages originated by small-scale allopatry in larger regions of relative stability^29^. Independent of habitat stability, some models consider species ranges a time-dependent dynamic attribute, so that narrow ranges are interpreted as a temporary characteristic of younger species^32^. Concerning lineage-specific or intrinsic factors, low dispersal ability and/or specialisation and associated phenotypic traits are typically invoked as explanations for microendemicity of species originated in allopatry^33–36^. Regardless of the actual extrinsic or intrinsic factors leading to speciation with an effect on species ranges, most microendemicity studies stress a correlation between microendemic species and allopatric ranges, deducing that allopatric speciation is a dominant explanation for the origin of microendemic biodiversity^29,32,37,38^. These same intrinsic and to some degree also extrinsic factors influence how organisms diversify through time^39,40^. Thus, the study of the tempo and mode of diversification is an important analytical tool to unravel the mechanisms leading to particular biogeographic patterns, including microendemicity^7,28,29,36,41,42^.

There have been some important recent contributions describing the diversity and distribution of the hyperdiverse Eumolpinae leaf beetles in one of the main microendemicity hotspots, New Caledonia^22^. Resulting from the improved taxonomic knowledge about these beetles, it was uncovered a generalised pattern of microendemicity for most species, generally known from a single or few geographically close localities, within a single valley or elevation^43–48^. These findings, concordant with reports for many other arthropod groups in the island^1^, prompted us to investigate the evolutionary mechanisms that could explain such a frequent outcome. Here, we will study these mechanisms in two endemic sibling genera of New Caledonian leaf beetles with similar distribution patterns in New Caledonia, *Taophila* Heller and *Tricholapita* Gómez-Zurita and Cardoso. Both genera consist of many species, most of them showing microendemic distributions^47,48^, and our main aim is establishing the general evolutionary process that led to the observed distribution patterns. Current knowledge on diversity and distribution patterns and the lack of temporal and spatial resolution about particular events that may or may not correlate with these patterns hinder addressing the general question why microendemicity evolves. However, we can shed some light on how it may originate in a particular group and region, as a first step towards a much-needed synthesis on this topic. Our strategy will involve the analysis of diversification in the clade of interest and an attempt to fit a model of speciation to the diversification process, in order to assess the degree in which geographic distributions and allopatry could be important factors responsible for the microendemicity patterns of these two New Caledonian leaf beetle genera.

## Results

### Phylogenetic backbone for diversification analyses

**Figure 1:**
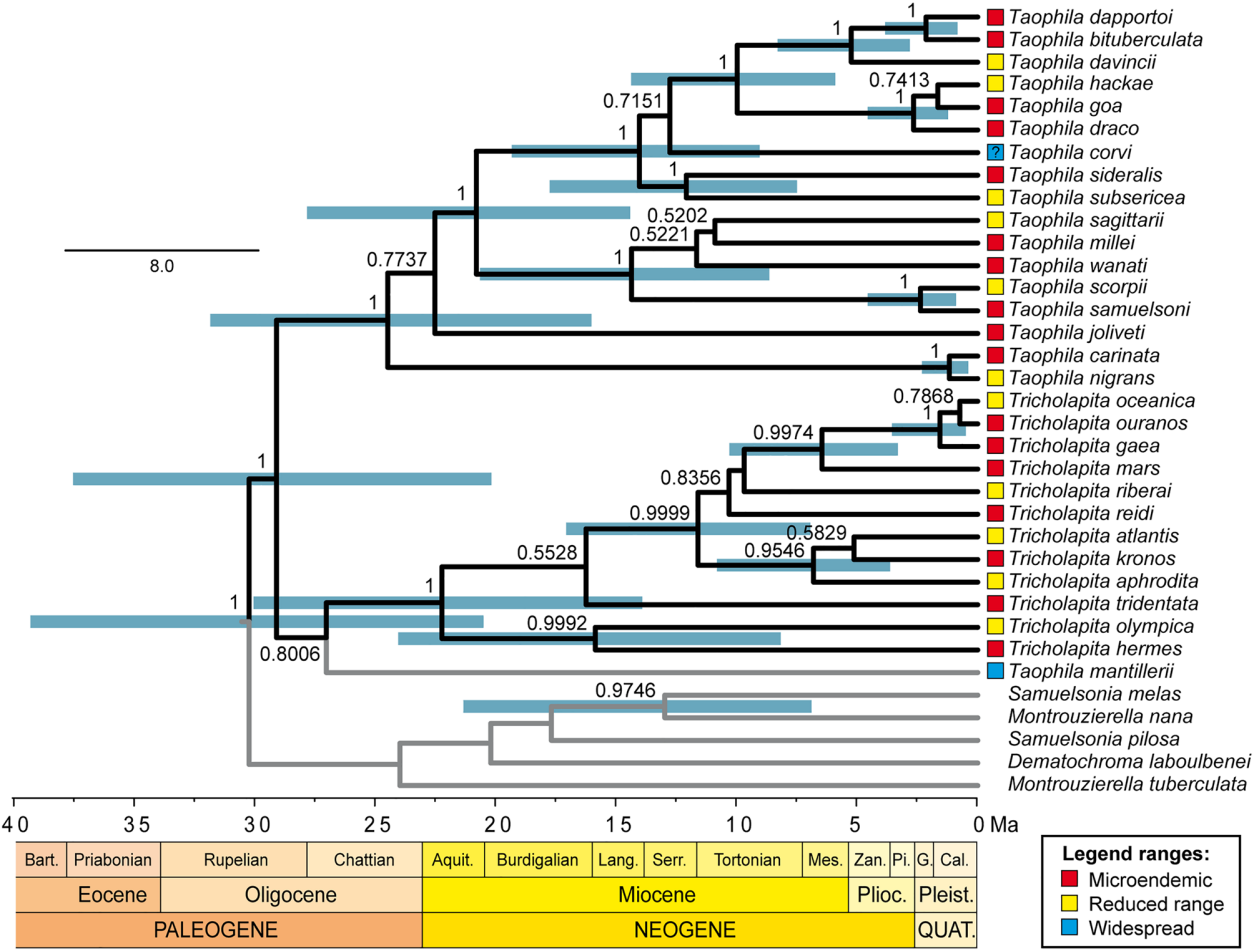
Uncorrelated-lognormal clock-constrained tree of New Caledonian Eumolpinae, with focal groups *Taophila* and *Tricholapita* highlighted. Moderately (posterior probability [PP] = 0.50–0.95) and highly (PP = 0.95–1.00) supported nodes are labelled and the HPD age intervals of the latter represented by bars for each of the nodes. Terminals colour-coded according to accompanying legend based on known species distributions, where “microendemic” refers to species known from a single or geographically close localities; “reduced range” to species found in a regional set of localities; and “widespread” to species found across Grande Terre.

A clock-constrained Bayesian tree based on *cox1* and *rrnS* data and calibrated based on the results of previous studies was obtained for 30 species of *Taophila* and *Tricholapita*. The tree was compatible with phylogenies published separately for each genus (Fig. 1), showing their separation during the Oligocene (29.09 Ma; 95HPD = 20.18–37.53 Ma) and their crown diversifications in the Oligocene–Miocene transition: 24.48 Ma (95HPD = 16.03–31.84 Ma) for *Taophila*, and 22.23 Ma (95HPD = 13.92–30.04 Ma) for *Tricholapita*.

### Diversification model of Taophila and Tricholapita

**Figure 2:**
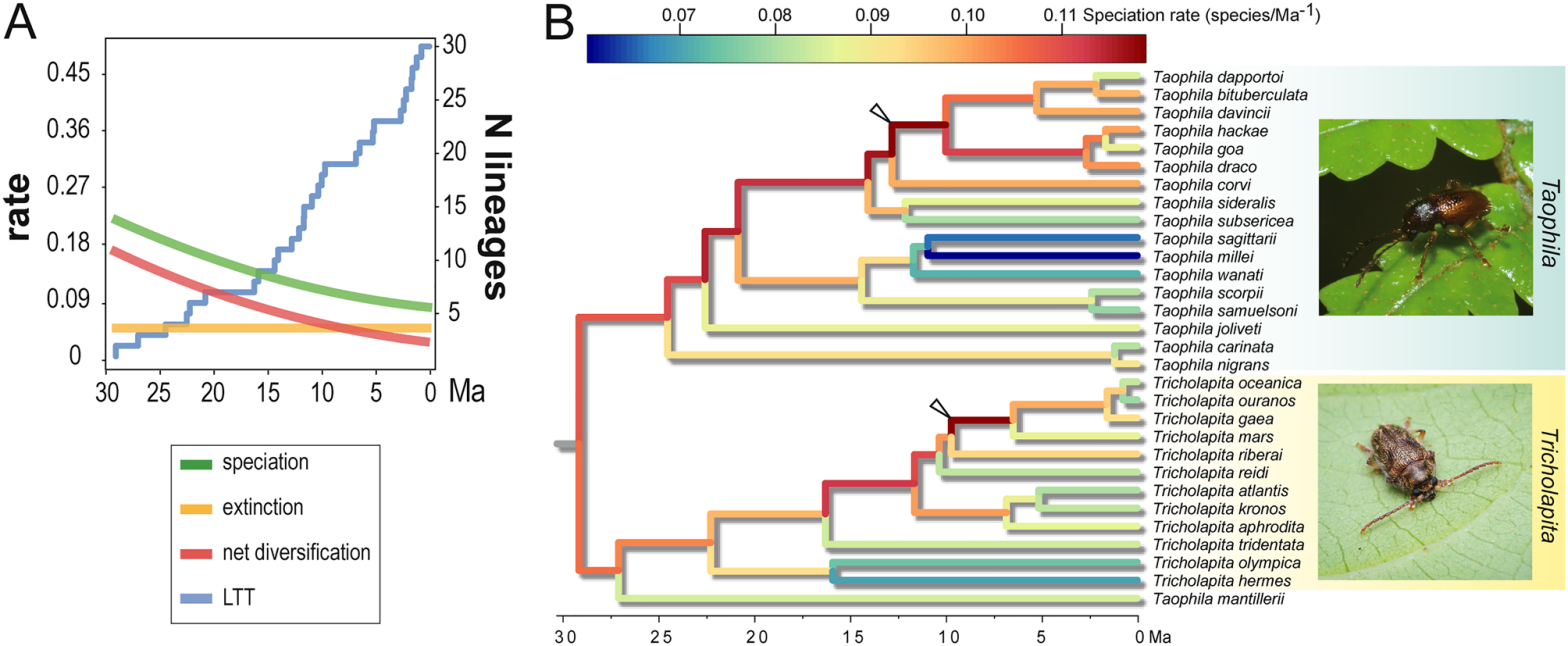
(**A**) Diversification-through-time and lineage-through-time (LTT) trajectories inferred using BAMM^104^ for the lineage of sibling genera *Taophila* and *Tricholapita*. Net diversification (red) decomposed in speciation (green) and extinction (yellow) components. Visual information on the trajectory of confidence intervals through time available in Fig. S1. (**B**) Branch-specific speciation rates in the evolution of *Taophila* and *Tricholapita*, with changes in tendency indicated by white arrowheads as obtained with the ClaDS model^109^. (Pictures obtained from iNaturalist and used with authors' permission: *Taophila*, Pierre-Louis Stenger; *Tricholapita,* Damien Brouste).

The analysis of global changes in evolutionary rates using BAMM showed that data fitted consistently a model of progressive slow-down of net diversification rates, owing to a gradual reduction in the rate of speciation against a relatively constant estimate of extinction rate (Figs. 2A and S2). Mean speciation and extinction rates were λ = 0.120703 and µ = 0.065015, yielding a mean net diversification rate of 0.055688 events/Ma.

Testing for shifts in diversification rates produced very low Bayes factor values (BF = 0.1922) for *k* = 1, and increasing values of *k* further decreased this BF value, approaching it to 0. Such low BF values indicate rejection of the alternative over the null hypothesis or, considering the relatively small size of the dataset, at least insufficient evidence to reject the null hypothesis of no shifts in diversification rate for the whole dataset. Alternatively, the analysis of local shifts in diversification rates with CLaDS co-estimated the values of initial speciation rate, λ_0_ = − 2.06214; factor controlling freedom of change of daughter speciation rates, σ = 0.237885; factor controlling increasing or decreasing trends at speciation, α = 0.919254; and constant turnover, ε = 0.128263. Accordingly, the mean relative change in rate at speciation in the ingroup phylogeny was m = 0.945635. For m < 1, data is consistent with a scenario of low heterogeneity of generally decreasing diversification rates, indicating that the chance of speciation diminishes, even if weakly, as diversification proceeds (as opposed to m > 1, indicating an acceleration in speciation). Figure 2B shows this trend graphically, only reversed in the clade of *Tricholapita mars* and relatives, and the clade including the most recent diversification of *Taophila*, around the species *Ta. draco* and *Ta. davincii*.

Up to twenty-one diversification models, including constant-rate models, time-dependent, temperature-dependent, diversity-dependent and protracted speciation models were tested for the data (Table 1). The model receiving the best AICc value was protracted speciation (PS21), with relatively large AIC-based distances (8.52 < ΔAICc < 14.65) compared with other models, irrespective of the correlated factor, i.e. time, temperature or diversity. The best-fitting model proved adequate for the phylogeny, with tree-shape metrics derived from simulations under this model statistically identical to metrics obtained from the empirical tree (Fig. S3): principal Eigenvalue = 3002.55 (*P* = 0.552); asymmetry = − 0.13967 (*P* = 0.926); and peakedness = 2.109148 *(P* = 0.980).

**Table 1:** Models of diversification for ingroup taxa, with empirical speciation (λ_0_) and extinction (µ_0_) rates, sign and rapidity of their variation (α and β, respectively), log-likelihoods and the respective AICc and Akaike weight (*w*) values. The best fitting model with the lowest ΔAICc is highlighted in bold. Models were tested with RPANDA [108] in most cases, except in the case of DD and PS models, which used DDD [111] and ProSSE functions [112], respectively.

Model	λ_0_	α	µ_0_	β	LH	AICc	*w*	ΔAICc
CR1	0.081879	NA	0	NA	− 99.4223	200.9874	0.013123	8.5199
CR2	0.081846	NA	0	NA	− 99.4223	203.2890	0.004207	10.8215
TD3	0.060911	0.029796	0	NA	− 98.7411	201.9266	0.008349	9.4591
TD4	0.058611	0.002536	0	NA	− 98.7554	201.9552	0.008300	9.4877
TD5	0.081951	NA	0	0.018322	− 99.4223	205.7676	0.001244	13.3001
TD6	0.081865	NA	0.000001	0	− 99.4230	205.7690	0.001245	13.3015
TD7	0.061324	0.030494	0.002369	NA	− 98.7410	204.4050	0.002465	11.9375
TD8	0.060633	0.003863	0.027279	NA	− 98.7494	204.4219	0.002450	11.9544
TD9	0.075387	0.024385	0.122112	0.333620	− 98.6967	206.9934	0.000679	14.5259
TD10	0.069481	0.006570	0.154848	0.014449	− 98.5590	206.7181	0.000780	14.2506
ED11	0.060060	0.037004	0	NA	− 98.7601	201.9646	0.008404	9.4971
ED12	0.052186	0.005396	0	NA	− 99.0436	202.5317	0.006383	10.0642
ED13	0.081887	NA	0	0.028909	− 99.4223	205.7676	0.001274	13.3001
ED14	0.081887	NA	0.000003	0	− 99.4230	205.7690	0.001275	13.3015
ED15	0.060240	0.036711	0	NA	− 98.7601	204.4433	0.002476	11.9758
ED16	0.052355	0.005371	0	NA	− 99.0437	205.0104	0.001870	12.5429
ED17	0.060077	0.036971	0.000002	0.025275	− 98.7601	207.1201	0.000652	14.6526
ED18	0.078107	0.020920	0.597715	0.092445	− 98.5617	206.7235	0.000796	14.2560
DD19	0.278675	NA	0.066119	NA	− 97.5925	203.1849	0.004673	10.7174
DD20	0.526296	NA	0.028105	NA	− 98.1920	204.3840	0.002578	11.9165
PS21*	**0.103939**	**NA**	** < 0.000001**	**NA**	**− 92.7538**	**192.4675**	**1**	**0**

*Models:* constant speciation (CR1) and constant extinction (CR2); exponential (TD3) and linear (TD4) time-dependence of speciation and no extinction; exponential (TD5) and linear (TD6) time-dependence of extinction and constant speciation; exponential (TD7) and linear (TD8) time-dependence of speciation and constant extinction; exponential (TD9) and linear (TD10) time-dependence of speciation and extinction; exponential (ED11) and linear (ED12) temperature-dependence of speciation and no extinction; exponential (ED13) and linear (ED14) temperature-dependence of extinction and constant speciation; exponential (ED15) and linear (ED16) temperature-dependence of speciation and constant extinction; exponential (ED17) and linear (ED18) temperature-dependence of speciation and extinction; exponential (DD19) and linear (DD20) diversity-dependence of speciation and constant extinction; and protracted speciation (PS21).

*The reported rate for this model is the speciation initiation rate. The protracted speciation model includes an additional parameter, speciation completion rate (λ) = 0.1401414, for our dataset*.*

### Speciation modes in Taophila and Tricholapita

The ingroup mode of speciation was tested explicitly by incorporating data on the phylogeny, age of lineages, and range overlap between species. Standard age-range correlation analyses in *Taophila* and *Tricholapita* were only significant for the intercept (*P* = 0.0184) and the slope (*P* = 0.0185) of the correlation for point distribution ranges in *Taophila* (and marginally significant for the slope [*P* = 0.0799] of buffer ranges in *Taophila*). Otherwise, the observed distribution of slopes and intercepts were not different from random (Fig. S4). In turn, the PGLS regression between log-transformed species ages and ranges, implementing Pagel’s λ = − 0.0102, was not significant (slope = − 0.056, *P* = 0.630; Fig. S5), rejecting the hypothesis of younger species having smaller ranges. ARC results were also one of the summary metrics among fourteen included in the process-based DReaD simulation model of^49^ and Table S3 shows the estimates of summary statistics by DReaD for both genera. The accuracy of model classifications was statistically significant (κ = 0.5752, *P* < < 0.001), indicating that the obtained classification was better than random. In these classifications, vicariant speciation dominated in *Taophila* (94.71%), followed by founder (4.05%) and mixed (1.21%) models. In *Tricholapita*, vicariant speciation was almost exclusive (98.94%), with a mixed model as the second alternative (1.06%).

## Discussion

We investigated the diversification dynamics and the geography of speciation of New Caledonian rainforest leaf beetle sibling genera *Taophila* and *Tricholapita*, characterised by mosaic-like microendemic distributions of most species. This pattern characterises many groups of organisms in New Caledonia and elsewhere, but we lack a general understanding of the conditions that lead to its development. Unveiling the mechanisms that result in these spatial patterns, and here we focused specifically on intrinsic evolutionary factors, may be crucial to understanding how microendemic diversity is generated, and more importantly how narrow species ranges are maintained, which is relevant for conservation^50,51^. The small size of our dataset, imposed by the limited diversity of the group of study, together with lack of fossil data, recommends much caution when interpreting results of diversification analyses owing to statistical power limitations of current analytical approaches, among others^52–54^. Thus, echoing the assessment by^55^, we only consider net diversification dynamics and avoid interpretations about speciation and extinction rates.

A suitable summary for the evolution of the *Taophila* and *Tricholapita* lineage generally fits a scenario of very small decline in diversification rate through time and of allopatric speciation as the dominant process explaining its diversity. Regardless of the net speciation/extinction inferred values, the diversification trend for this lineage is suggestive a priori of a reduction in diversification rate. The progressive slowdown in diversification rates is a common evolutionary trend^56–60^, and it has been reported for several organisms in New Caledonia^61^, including the entire diversification of Eumolpinae^22^. There are several accepted evolutionary scenarios that could lead to a reduction in diversification rates^59^, which fall into three categories, including (i) diversity-dependent, (ii) time-dependent, and (iii) protracted speciation, the latter implying that species are not immediately recognisable as separate genetic entities at the tips of a phylogeny^62^. Data for *Taophila* and *Tricholapita* were consistent with the latter, indicating that if a rate slowdown truly describes the diversification of this lineage, as hinted with different methods (Table 1; BAMM Figs. 2 and S1 and CLaDS Fig. S6), it is most likely related to topological conditioning of gene trees, and to the prolonged nature of the speciation stages rather than an actual trend in the diversification process. Protracted speciation implies that terminal branch lengths in a species tree may be overestimated owing to potential incipient species not being considered in the tree^63^. Connecting this evolutionary pattern with the diversification of a group of organisms with microendemic ranges can be difficult, since microevolutionary processes leading to speciation in large, widespread populations are expected to be rare in this system. On the other hand, limited ranges and the logical difficulties for local differentiation within a small range could potentially lead to a phylogenetic pattern consistent with protracted speciation, especially if we take into account the apparent independence of species age with their range size, implying that the lack of microevolutionary differentiation may be persistent in time. For the sake of discussion, consistent failure to sample sister species of represented lineages in the phylogeny may yield longer than average terminal branches too, but this bias seems unlikely considering our recent taxonomic revisions^46–48^. Fitting the protracted speciation model may be more suitable for datasets based on population data and not species trees, and perhaps larger datasets where estimation of parameters may be less compromised. However, in the case of microendemic insect species with ability to disperse, effective panmixia and the absence of local genetic differentiation may be common, limiting the potential of lineage-based diversification analyses. Reduced, sometimes absent mtDNA haplotype diversity in the case of *Taophila*^48^ and *Tricholapita*^47^ would support this view.

The other correlative models considering time, temperature or diversity, as well as null, constant rate models, proved clearly inferior to protracted speciation for this dataset. These other models loosely clustered according to their number of free parameters, suggesting that the result of model selection could be influenced by the lack of penalty for estimating some of the parameters, e.g. extinction. However, the overall best fit of protracted speciation to the dataset, particularly with the previous considerations about microendemic distributions, does not imply that the other processes do not operate on this system, but possibly that there are statistical limitations to recognise their effects or that their effects do not lead to rate slowdowns consistently across the phylogeny^63^. Moreover, disentangling these effects can be challenging, even for larger datasets^64^. Time-dependent diversification is often connected to environmental change^59^, for example through a correlation between declines in speciation and gradual cooling of global temperature^65^, or considering the effect of past climatic stability on niche partitioning^60,66^. To complicate things even further, environmental effects, or more specifically the influence of temperature can potentially mimic the outcome of diversity-dependent processes too^60,64,67,68^.

There are multiple ways in which these interconnected processes can exert non-directed or rate-declining influence on the diversification of New Caledonian Eumolpinae, also contributing to reduction of ranges. Global cooling and episodic climatic events, with periods of aridification that reduced the primary forest in Grande Terre, may have reduced suitable niches and species ranges^69–72^. Similarly, the geomorphological evolution of Grande Terre transitioned from a period of relative intensity during the mid-Oligocene^73,74^ to relative geological stability, dominated by erosion and planation, with an exceptionally active period during the Pleistocene^74,75^. This geological transition, concurrent with the evolution of *Taophila* and *Tricholapita*, could represent a trend from higher to lower topological and ecological complexity, potentially correlated with a reduction in diversification rates. The idea of a time-dependent process in New Caledonia somewhat associated to niche-filling, i.e. successive colonisation and preemptive occupation of empty niches^56,57,59,76,77^ further constraining the occupation of space, receives some support from the rate-decline exceptions of the fine-grain CLaDS analysis of changes in diversification rates across the phylogeny. One in particular affects the *Tricholapita* clade including the species *Tr. reidi*-*Tr. oceanica* (see Fig. 2B). This derived group of *Tricholapita* colonised and remained restricted to humid forests in southern parts of Grande Terre characterised by ultramafic soils, soils with high metallic content and consequently highly specialised vegetation^47,78^. Both host-plant choice and the intimate association of subterranean Eumolpinae larvae with the soil make it plausible that this lineage colonised and underwent an irreversible adaptation to ultramafic soils too. Colonising and exploiting a new, previously unoccupied niche could have released diversity-dependent diversification constraints and trigger a secondary wave of diversification and speciation in this group, with the successive fragmentation of parental ranges leading to current microendemic distributions.

The previous fundamentally ecological rules only explain in part the occupation of space, but not the drivers of speciation in these genera. Knowledge about the ecology of the species of *Taophila* and *Tricholapita* is very limited, aside from field observations of some species associated with ferns and DNA-based inferences on potential host plants in different botanical families^43^. We cannot rule out entirely an ecological driver for the origin of new species in these genera, for example by specialisation to different host plants. However, allopatric distributions of most species and documented cases of sympatry only for species that built enough phylogenetic and evolutionary divergence^48^ strongly suggest that some form of geographic speciation is at play for these genera^59,79^.

Disentangling the processes leading to speciation is at the core of many evolutionary studies^80^, but in the case of geographic speciation, the changing nature of species ranges and epistemological problems in establishing ancestral species ranges have jeopardised the reliability and statistical significance of the inferred processes^81,82^. Nonetheless, the interest in the geography of speciation spurred the development of several analytical approaches combining distribution and phylogenetic data^81,83–86^. Recently^49^, proposed a simulation and model-selection approach designed to infer speciation modes by simultaneously analysing multiple statistics representing different aspects of the interplay between speciation, phylogeny and current species ranges, typically examined individually in most other methods. Using this approach, vicariant speciation stood out as the dominant inference for *Tricholapita* and *Taophila*, the latter also with secondary signs of dispersal speciation. Allopatric speciation is the main explanation in most cases of microendemic distributions^37,87,88^, and it has been repeatedly invoked as the most likely speciation mechanism for New Caledonian microendemic species^32,61^. However, in previous studies, this process was considered an ad hoc hypothesis to explain certain patterns, and ours would be the first where this process is inferred analytically a priori for New Caledonian microendemic organisms. Nonetheless, invoking allopatric speciation as the mechanism generating microendemic diversity has theoretical requirements that need to be considered to provide a satisfactory explanation for the observed pattern^37^. The mechanism requires that the range of widespread ancestral species be fragmented by vicariance, by the formation of dispersal barriers, effectively isolating daughter populations^89^.

The ragged orography of Grande Terre was formed through successive cycles of planation during the Miocene and as recently as the pre-Pliocene, when glacio-eustatic processes might have been involved in considerable land tilting and uplift, followed by deep river erosion and valley formation^73,74^. These processes were contemporaneous with the diversification of *Taophila* and *Tricholapita* and in the latter the mechanism is compatible with the vicariance succession hypothesised for basal splits in the genus^47^. They also offer ample opportunity for cycles of species range expansions and successive range-reduction vicariance events in line with the evolution of *Taophila* or derived splits in both genera^48^. In support of this idea, there are some species in these genera that currently show relatively large ranges and also some phylogeographic evidence suggesting they have the potential for range expansion, as deduced for *T. subsericea* along the northern slopes of Massif du Panié^47,48^. In turn, elevation gradients favour habitat heterogeneity and local insular environments which, in combination with regional climatic changes during the past 25 Ma, could have generated the conditions for successive cycles of habitat fragmentation and contributed to subsequent allopatric speciation in many organisms adapted to the rainforest. Specifically, the late Miocene represented a relatively warm period within the general cooling trend that characterised most of the Cenozoic, and responsible for dramatic changes in ecosystems globally, usually resulting in cycles of aridification in lower latitudes^65^. In New Caledonia, it is assumed that aridification fragmented and contracted the humid forest in refugia available thanks to landscape heterogeneity, either higher elevations or areas of microclimatic suitability^69–72^. Climate-driven habitat fragmentation could contribute additional vicariance events also in periods of geological stability and establish conditions that could favour speciation, including range contractions and reduction in population size^90–92^. Although a marginal inference from our analyses, speciation following dispersal cannot be entirely discarded at least in the case of the genus *Taophila*, implying that some ancestral species could have expanded their range across preexisting barriers with subsequent speciation.

**Figure 3:**
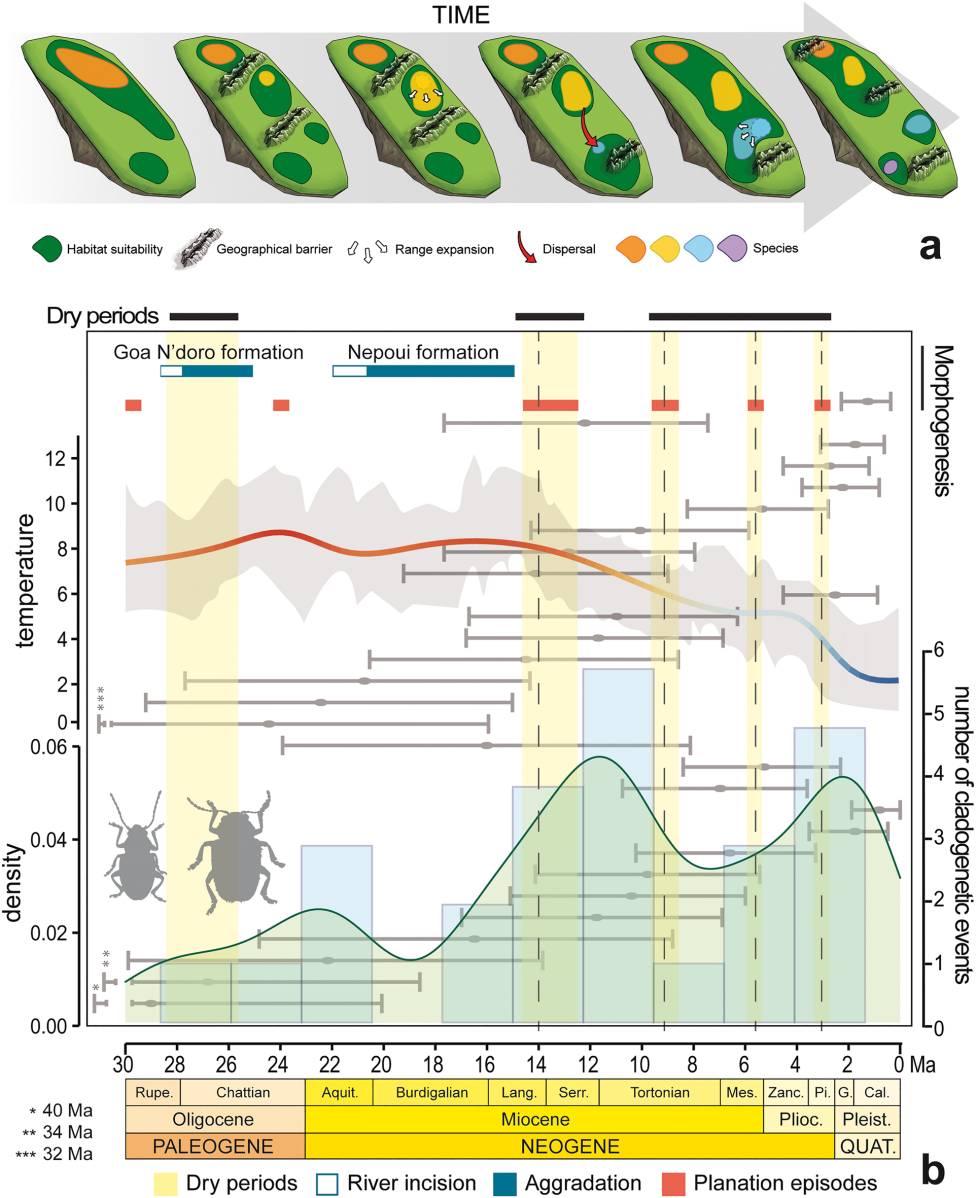
(**a**) Cartoon summary of the main spatial processes explained in the main text conducing to gradual occupation of habitat through successive speciation events and confinement to microendemic ranges in a scenario with reduced effects of extinction. (**b**) Histogram of the succession and variation in the number of phylogenetic splits along the evolution of *Taophila* and *Tricholapita* since the Oligocene, with gray horizontal bars showing confidence time intervals for these splits (shown in no particular order), together with contemporaneous palaeoenvironmental changes in temperature and precipitation regimes (pale yellow intervals and black bars on top) and major chronological periods of geomorphic evolution of Grande Terre (white, blue and red intervals), both based on the synthetic model of^74^.

An alternation during most of the Late Cenozoic of phases of relief formation and/or habitat fragmentation with periods dominated by erosion and/or habitat expansion in New Caledonia could explain in part the cycles of species range expansions, isolation of populations through vicariance and eventual speciation in allopatry with ever contracting ranges (Fig. 3a). But this alone is not sufficient to explain the accumulation of species with reduced ranges, and the concourse of environmental effects may have been required as well. The interplay of geological and environmental drivers can be hypothesised based on the apparent match of peaks of cladogenesis following dry periods, in turn responsible for the fragmentation of New Caledonian rainforests (Fig. 3b). The species surviving these geological and climatic cycles could have a limited potential for range expansions because of neighbouring species through some form of density-dependent processes, such as competitive exclusion^93,94^, perhaps reinforced by other negative biotic interactions, including reproductive interference^95^. This model would explain the gradual build-up of species diversity attaining microendemic distributions through the combined effects of isolation and negative biotic interactions. The idea that microendemicity reflects the maturity of a system approaching carrying capacity, contrasts with alternative hypotheses where microendemicity is associated with initial stages of speciation prior to species range expansions^32^. Indeed, most sister species pairs in our analysis have ranges of different size (Figs. 1 and S7) and the lack of a significant association between species age and range also argue against a pure effect of time alone on these ranges. Moreover, the model does not conflict with the possibility of sympatry, a pattern observed in several instances of *Taophila* and *Tricholapita*, which could have been attained secondarily when phenotypic and ecological differences between neighbouring species relaxed competition and other negative interactions. Secondary sympatry for these genera seems particularly frequent in few high elevation spots throughout the island, such as Aoupinié or Mandjélia^47,48^. This distribution pattern could be expected if these areas acted as climatic refugia during the Pleistocene and following the contraction of the rainforest as proposed for other organisms^32,71^.

The study of diversification is challenging for small clades, lacking the analytical power to provide definitive answers. However, the knowledge gained about possible modes of diversification in these cases can be at least and with every caution a starting point towards informed hypotheses for a better understanding of their diversity^55^. The study of diversification and speciation of the New Caledonian radiation of *Taophila* and *Tricholapita* revealed there is nothing extraordinary or unexpected about the putative intrinsic processes that led to their current microendemic species distributions. But their analytical characterisation is essential to incorporate these processes as ad hoc elements of an eventual explanation of spatial patterns. Our full understanding of the causes of microendemicity shall benefit from integrating micro- and macroevolutionary processes and explicit knowledge about both intrinsic and extrinsic factors, as illustrated by geological and climatic evolution matching cladogenesis in this group, how speciation occurred and what was the fate of the daughter species relative to inherited area and environmental conditions.

## Methods

### Study groups

Studies on microendemicity can be biased because of sampling artifacts^1^ and incorrect taxonomies^96^. Here, we selected two endemic sibling genera of New Caledonian Eumolpinae where these potential drawbacks are minimised: *Taophila* and *Tricholapita*. These genera were revised recently using material from our 2007–2008 research collection (IBB-CSIC, Spain) and Prof. M. Wanat's collection (Museum of Natural History of Wrocław University, Poland), obtained through intensive sampling of phytophagous beetles in New Caledonia between 2004 and 2010. Establishing species ranges mainly depends on sampling effort^97–99^. Admittedly, exploring new localities in New Caledonia, where much of the island’s wilderness is inaccessible, could potentially refine our knowledge both on species diversity and distributions. However, our current ideas about species ranges and boundaries seem consistent with information for other groups^100^, representing a relatively solid starting point to start questioning about the relationship between species ranges and evolution. *Taophila* consists of 21 species, 18 of which show reduced, mostly allopatric ranges, 14 of them known from a single locality^48^. In turn, *Tricholapita* includes twelve species, all with restricted and usually allopatric ranges and seven known from a single locality^46,47^. Relevant genetic data were available for all species of *Tricholapita* and 18 out of 21 species of *Taophila* (Table S1).

### Clock-constrained phylogeny

We used mtDNA gene trees based on partial cytochrome *c* oxidase subunit 1 (*cox1*) and small rRNA subunit (*rrnS*) genes as a proxy to *Taophila* and *Tricholapita* species phylogenies (most source material were dry collection specimens, which failed to yield nuclear gene PCR products). In these genera, mtDNA data alone were reliable for species delimitation and relationships, with nearly perfect matches between taxa and species monophyly deduced from multiple individuals, a single instance of incongruence due to interspecific hybridisation between locally sympatric species^48^, and species phylogenetic clusters compatible with morphological species groups^47,48^. Representative sequences from one individual per species as well as several outgroups (Table S1; Fig. 1) were obtained from published studies^22,43,46–48^. Sequences were aligned for each marker separately using the G-INS-i algorithm in MAFFT 7.3^101^, and concatenated in a single alignment to infer a time-constrained ultrametric tree in BEAST 1.8.4^102^. Data were analysed using unlinked^103^ evolutionary models for each marker, with site-heterogeneity in the rates of evolution, including a proportion of invariable sites, nucleotide frequencies estimated from the sequences, a Birth–Death speciation tree model and uncorrelated lognormal relaxed clocks for each marker. The tree was time-calibrated using two calibration points derived from clade ages inferred by^22^, who used the same phylogenetic markers and calibration hypotheses based on either marker-specific rates or on biogeographic considerations that yielded highly consistent age estimates. Specifically, we used rate-based inferences for the stem age of *Tricholapita*, dated at 32.6 Ma (95% Highest Posterior Density [95HPD] = 26.2–39.9 Ma), and the crown age of “Clade I” in^22^, including representatives of the groups of *Ta. sagittarii* and *Ta. subsericea*^48^, dated at 26.3 Ma (95HPD = 10.9–31.9 Ma). These two calibration nodes were modelled to cover the respective 95HPD confidence intervals as normal distributions with means 33.0 and 22.0, and standard deviations of 4.0 and 6.5, respectively. Bayesian inference used a MCMC search with 75 million generations, sampling trees and parameters every 7500 steps, and final estimation of parameters and maximum clade credibility tree were obtained after discarding 10% of the initial results.

### Diversification analyses

The time-constrained tree topology was used to study different aspects of diversification in *Taophila* and *Tricholapita*. Potential heterogeneity in evolutionary rates through time was studied based on Bayesian Analysis of Macroevolutionary Mixtures (BAMM^104^). BAMM analyses were run for the whole ingroup dataset (n = 30) and the speciation-extinction test consisted of 20 million simulation steps, sampling data every 1000th generation, estimating taxonomic coverage as the fraction of available taxa from the known species catalogues, with additional priors (Table S2) obtained using the function setBAMMpriors in the R package BAMMTools 2.0.2^105^. Global rate shifts were tested with BAMM considering models with up to *k* = 5 shifts, using Bayes Factors to compare against a null model without shifts in net diversification rate. These analyses make inferences about the diversification process considering global rates and major shifts across phylogenies, relying on strong assumptions about species representation in the sample^106^. We alternatively implemented a Bayesian approach that analyses branch-specific changes in diversification rates at each speciation event under a birth–death diversification process, the ClaDS model^107^, as implemented in the RPANDA 1.9 package^108^. This approach complements some of the sample assumptions of the standard BAMM approach by explicitly modelling hidden speciation events, e.g., events missing in the phylogeny either because of extinction or sampling issues^60^. The Julia language implementation of the ClaDS model^109^ used here considers a scenario with variable extinction rate across lineages modelled by a constant turnover parameter, proven superior to other potential alternatives, such as excluding extinction or considering a constant extinction rate across lineages^107^. Finally, we evaluated the fit of the deduced diversification process to different diversification models using a maximum-likelihood framework, testing for potential effects of biotic (e.g., competition) or abiotic (e.g., temperature) factors on this rate. Specifically, we tested 21 models (see Table 1 for details), including two constant-rate models (pure-birth, CR1; birth–death, CR2), eight models with rates varying through time (pure-birth, TD3–4; birth–death, TD5–10), eight temperature-dependent models (pure-birth, ED11–12; birth–death, ED13–18), two diversity-dependent models with constant extinction (DD19–20); and protracted speciation (PS21). Time- and temperature-dependent models were fitted using RPANDA 1.9^108^, integrating palaeotemperature data derived from δ18O measurements from^110^ equations; diversity-dependent models using DDD 4.4^111^; and protracted speciation using ProSSE functions^112^ as implemented in the package diversitree 0.9.16^113^, which is the most robust current approach to reduce the error boundary in parameter estimates for this model in species trees when lineage-level phylogenies are not available^112^. Best-fitting models were selected based on differences of AICc estimates. The relatively small size of the dataset could impair the ability of these tests to accurately discern alternative diversification models^39^. Therefore, as a safeguard for model selection, we performed adequacy tests of best-fitting models using the R package BoskR^114^, which assesses shape similarity between empirical trees and a set of trees simulated under the tested model based on metrics derived from the tree Laplacian spectrum, interpreting uncorrected (to avoid inflation of type I errors^115^) significant differences as model inadequacy^116^. The simulation of protracted speciation is not implemented in BoskR, thus 1000 trees were simulated using the tree.prosse function of the diversitree package, fixing the number of tips, maximum root age and diversification rate to match the values deduced from the original tree, and subsequently used as input for the BoskR analysis.

### Speciation analyses

Microendemicity is a biodiversity pattern defined by geographic distributions, and may or may not be the result of the actual speciation process that generated the microendemisms. We explored explicitly the geography of speciation in *Taophila* and *Tricholapita* using standard age-range correlation (ARC) analyses, specifically testing the range overlap between species^81^ using the enmtools.aoc function in the R package ENMTools 1.0.1^117^ and two different measures of species ranges, considering point localities, as proposed by^83^, or fixed-radius buffered ranges. Specifically, these ranges considered circular buffers with radius = 8 km around data points (following, e.g.,^32^) obtained from geographic coordinates of occurrence data^46–48^, calculated and transformed into raster data on a 0.5-min resolution grid with ENMTools. We analysed another temporal aspect of species distributions, addressing the question whether recent speciation events could be associated to smaller ranges. This was tested through phylogenetic generalised least squares (PGLS) regression between terminal branch ages in the dated phylogeny and species ranges as deduced from the raster data. The PGLS regression was studied using the gls function of the nlme package in R^118^, based on an estimation of Pagel’s^119^ lambda. A preliminary analysis of the original variables showed a certain degree of positive skewness (Fig. S1), thus final analyses used the logarithmic transformation of the variables. The analysis used the full dataset after checking for outliers based on potential departures of homoscedasticity and normally-distributed residuals.

The model of speciation best fitting the phylogeny and species geographic ranges was inferred using a process-based simulation model that considers 14 summary metrics related to the speciation modes under the assumption supported by simulations that phylogenetic and geographic patterns retain signals of the history of speciation^49^. The model allows distinguishing among five speciation modes, including allopatric or vicariant, sympatric, parapatric, by dispersal and founder events, and a mixed mode including the previous four. The analysis of speciation mode used the phylogenies for each genus separately, and species ranges estimated as above, and it was run with the set of functions and the DReaD simulation established by^49^. This analysis included inference of summary metrics relevant for discrimination of speciation modes, likelihood-free model selection based on machine-learning linear discriminant analysis (LDA) and cross-validation accuracy test for model classification, and tests were performed with the R packages klaR 0.6-15^120^ and caret 6.0^121^. Here, LDA model selection assigns posterior probabilities to each model class to rank their potential contribution to the observed pattern.

All the statistical analyses described were run in the R environment^122^ (or the Julia environment^123^, when indicated).

## Supplementary Information

Supplementary Information 1.

Supplementary Information 2.

Supplementary Information 3.

## Data Availability

All the sequences and distribution data used for this study are publicly available as data associated to articles Papadopoulou et al*.* (2013), Gómez-Zurita and Cardoso (2014), Platania et al*.* (2020), Gómez-Zurita et al*.* (2020) and Platania and Gómez-Zurita (2022), and are under sequence accession numbers of the European Nucleotide Archive (https://www.ebi.ac.uk/ena/browser/home) listed in Table S1 of the Supplementary Information. Scripts and data files used for the analyses are included as part of the Supplementary Information of this article.
