# Supplementary material for: Analysis of intrinsic evolutionary factors leading to microendemic distributions in New Caledonian leaf beetles
Source: Sci Rep. 2023 Apr 27;13:6909. doi: 10.1038/s41598-023-34104-z (PMC10140066; doi:10.1038/s41598-023-34104-z)
Supplement: Supplementary file 1 — Supplementary Information 1. [file 41598_2023_34104_MOESM1_ESM.pdf]

Supplementary Information

Analysis of intrinsic evolutionary factors leading to microendemic distributions in New Caledonian leaf beetles

Leonardo PLATANIA & Jesús GÓMEZ-ZURITA

Table S1. Samples and sequence data used to infer the phylogenetic tree for diversification analyses.

Table S2. Values of parameters used in the BAMM analyses (value of the parameter `expectedNumberOfShifts` = 1.0).

Table S3. Summary metrics of the analysis of the geographic mode of speciation in *Taophila* and *Tricholapita* using DReaD (Skeels & Cardillo 2019).

Figure S1. Relationships between native values for species age and species range size established under PGLS testing considering the effects of phylogeny based on the correlation with Pagel's lambda (a). Analysis of data heteroscedasticity based on the distribution of normalised residuals versus fitted values (b) and quantile-quantile plots of normalised residuals to detect departures from normally-distributed residuals (c). Frequency distribution of residuals from the PGLS regression of age and range values correlated with Pagel's lambda (d).

Figure S2. Diversification-through-time trajectories decomposed as speciation (green), extinction (black) and net diversification (red), and their confidence intervals as inferred for the whole ingroup (a), as well as for *Taophila* (b) and *Tricholapita* (c) separately. Ingroup phylogenetic tree showing global trend in decrease of diversification rate as deduced with BAMMTools.

Figure S3. Results of adequacy analysis for the protracted speciation model, the best model fitting the ingroup phylogeny of *Taophila* and *Tricholapita*. Plots show simulated (density curve) and empirical (vertical line) values of relevant tree metrics, including principal Eigenvalue (a), asymmetry (b) and peakedness (c). Description of models in Table 1 of the main text.

Figure S4. Age-Range correlation tests in *Taophila* (a, b) and *Tricholapita* (c, d), obtained considering either ranges (a, c) or points of distribution (b, d). Scatterplots show the distribution of topologically averaged overlaps and their regression (black line) for empirical data, and the distribution of linear regressions from the Monte Carlo replicates (grey lines). Histograms represent the distribution of slopes (center panels) and intercepts (right panels) from the Monte Carlo replicates, and the black dashed lines show the values obtained from the empirical data.

Figure S5. Relationship between log-transformed values for species age and species range size established under PGLS testing corrected based on the correlation with Pagel's lambda (a), and analysis of data heteroscedasticity based on the distribution of

normalised residuals versus fitted values for this analysis (b) and quantile-quantile plots of normalised residuals to detect departures from normally-distributed residuals (c).

Figure S6. Diversity through time (a) and global rate through time (b) and their confidence intervals estimated from the ingroup tree of *Taophila* and *Tricholapita* in CLaDS analysis using Markov Chain Monte Carlo iteration.

Figure S7. Distribution maps of sister species or groups of three closest relatives in *Taophila* Heller and *Tricholapita* Gómez-Zurita & Cardoso: *Ta. bituberculata, Ta. dapportoi* and *Ta. davincii* (a); *Ta. draco, Ta. goa* and *Ta. hackae* (b); *Ta. sideralis* and *Ta. subsericea* (c); *Ta. millei, Ta. sagittarii* and *Ta. wanati* (d); *Ta. samuelsoni* and *Ta. scorpii* (e); *Tr. gaea, Tr. oceanica* and *Tr. ouranos* (f); *Tr. aphrodita, Tr. atlantis* and *Tr. kronos* (g); and *Tr. hermes* and *Tr. olympica* (h). Specimen collection data were plotted on maps using ggplot2 3.3.5 (Wickham, 2016), elevatr 0.4.2 (Hollister et al., 2022) and Natural Earth resources (https://www.naturalearthdata.com/) in R 4.1.2 (R Core Team, 2021).

References

Hollister, J., Shah, T., Robitaille, A. L., Beck, M. W., Johnson, M. (2022). elevatr: access elevation data from various APIs. https://github.com/jhollist/elevatr.

R Core Team. (2021). R: a language and environment for statistical computing. R Foundation for Statistical Computing, Vienna, Austria. https://www.R-project.org/.

Skeels, A., Cardillo, M. 2019. Reconstructing the geography of speciation from contemporary biodiversity data. Am. Nat. 193, 240–255.

Wickham, H. (2016). ggplot2: Elegant Graphics for Data Analysis. Springer-Verlag New York. https://ggplot2.tidyverse.org.

Table S1. Samples and sequence data used to infer the phylogenetic tree for diversification analyses.

Species	Voucher	*cox1*	*rrnS*	Range[a]
Taophila s. str.				
T. bituberculata	NC102	HF922224	HF921454	M
T. carinata	4384	OA984990	OA985053	M
T. corvi	4605	OA984991	OA985058	W?
T. dapportoi	4452	OA984993	OA985059	M
T. davincii	4397	OA984995	OA985062	R
T. draco	4407	OA984997	OA985065	M
T. goa	4420	OA984998	OA985066	M
T. hackae	NC083	HF922214	HF921444	R
T. joliveti	4601	OA985002	OA985068	M
T. millei	4594	OA985006	-	M
T. nigrans	4381	OA985007	OA985072	R
T. sagittarii	NC060	HF922205	HF921435	R
T. samuelsoni	4380/4413*	OA985011	OA985075	M
T. scorpii	4607	OA985012	OA985076	R
T. sideralis	4599	OA985016	OA985079	M
T. subsericea	4460	OA985020	OA985082	R
T. wanati	4596	OA985025	-	M
Taophila (Jolivetiana)				
T. mantillerii	4373	OA985003	OA985069	W
Tricholapita				
T. aphrodita	3939	LR634002	LR633983	R
T. atlantis	3950	LR634006	-	R
T. gaea	NC277	HF922173	HF921400	M
T. hermes	3941	LR634008	LR633987	M
T. kronos	3932	LR634009	LR633989	M
T. mars	NC058	HF922197	HF921427	M
T. oceanica	3951	LR634013	LR633994	R
T. olympica	3949	LR634014	LR633995	R
T. ouranos	3983	-	LR633996	M
T. reidi	3952	-	MT968974	M
T. riberai	3940	LR634015	LR633997	R
T. tridentata	4373	-	LR633999	M
**Outgroups**				
Dematochroma laboulbenei	NC855	HF921742	HF920954	-
Montrouzierella nana	NC711	HF921857	HF921071	-
Montrouzierella tuberculata	NC661	HF921961	HF921175	-
Samuelsonia melas	NC786	HF922127	HF921351	-
Samuelsonia pilosa	NC816	HF922139	HF921367	-

[a]Microendemic range (M), reduced range in nearby localities (R), and widely distributed in Grande Terre (W).
*The combined sequence is a chimera.

Table S2. Values of parameters used in the BAMM analyses (value of the parameter `expectedNumberOfShifts` = 1.0).

**Parameter**	**Ingroup**
`globalSamplingFraction`	0.90909
`lambdaInitPrior*`	2.14858227
`lambdaShiftPrior`	0.03957373

*The same values for `muInitPrior`.

Table S3. Summary metrics of the analysis of the geographic mode of speciation in *Taophila* and *Tricholapita* using DReaD (Skeels & Cardillo 2019).

Summary metric	*Taophila*	*Tricholapita*
Age-range correlation, slope	0.079896	0.291635
Age-range correlation, intercept	0.171441	0.320034
Mean RO	0.316984	0.034755
Proportion of sister species with RO ≥ 0.9	0.333333	0
Proportion of sister species with RO = 1.0	0	0
Mean of differences between RO and range overlap of each species with outgroup	0.185078	-0.352390
Slope of regression [RA x divergence time]	-0.337738	0.113119
Intercept of regression [RA x divergence time]	0.366877	0.390231
RS mean	0.282702	0.379867
RS standard deviation	0.233156	0.278915
Standard deviation of SDi between ranges of sister species	0.113643	0.405173
Intercept of regression SDi x divergence time	0.146424	-0.128350
Sackin's index	94	58

RO = geographic-range overlap between sister species; RA = range asymmetry (size differences between range of sister species); RS = range size; SDi = standardized distance (minimum distance between sister species ranges / maximum distance between species in the clade).

Figure S1

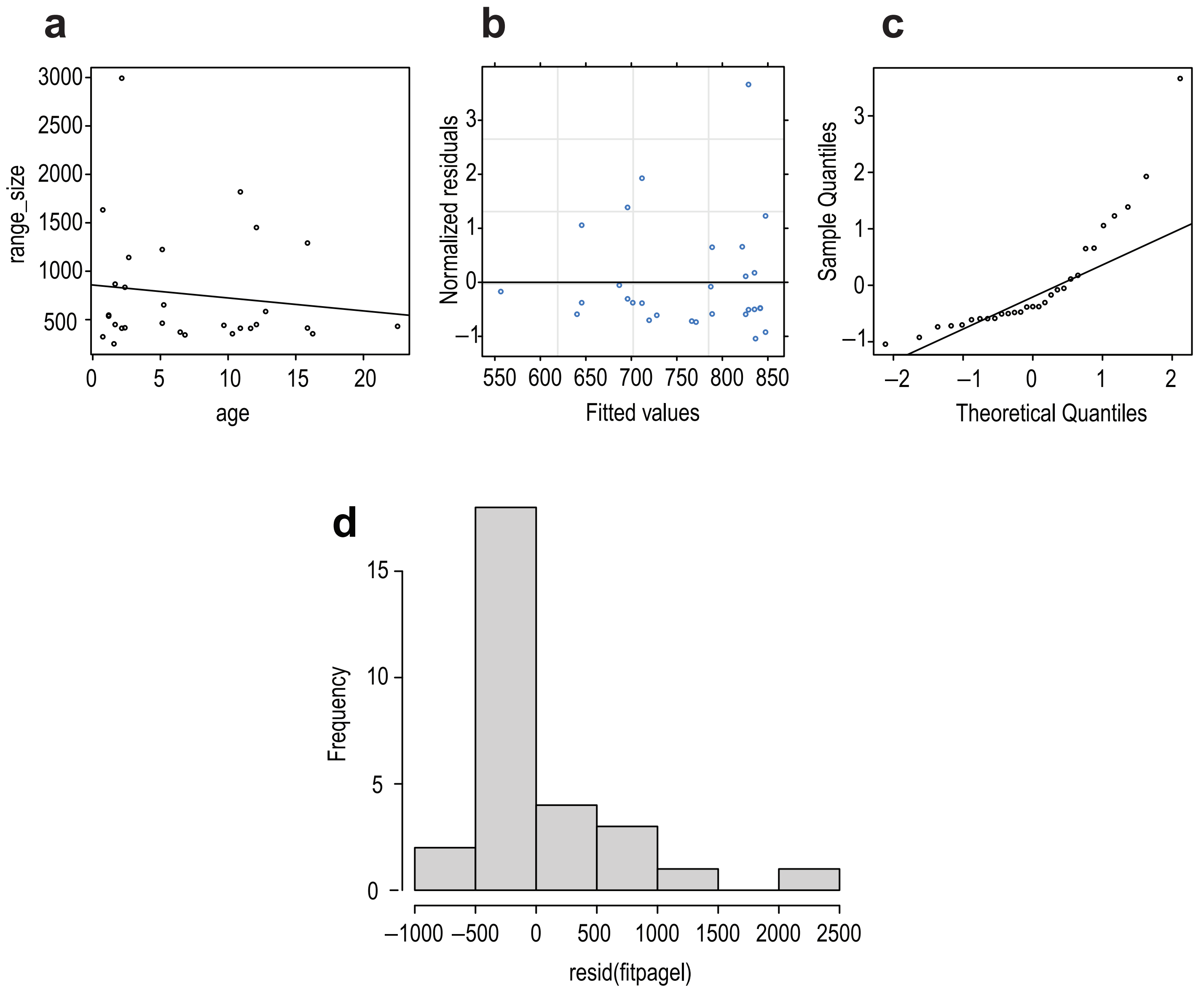
a
range_size
3000
2500
2000
1500
1000
500
0
5
10
15
20
age
b
Normalized residuals
3
2
1
0
−1
550
600
650
700
750
800
850
Fitted values
c
Sample Quantiles
3
2
1
0
−1
−2
−1
0
1
2
Theoretical Quantiles
d
Frequency
15
10
5
0
−1000
−500
0
500
1000
1500
2000
2500
resid(fitpagel)

Figure S2

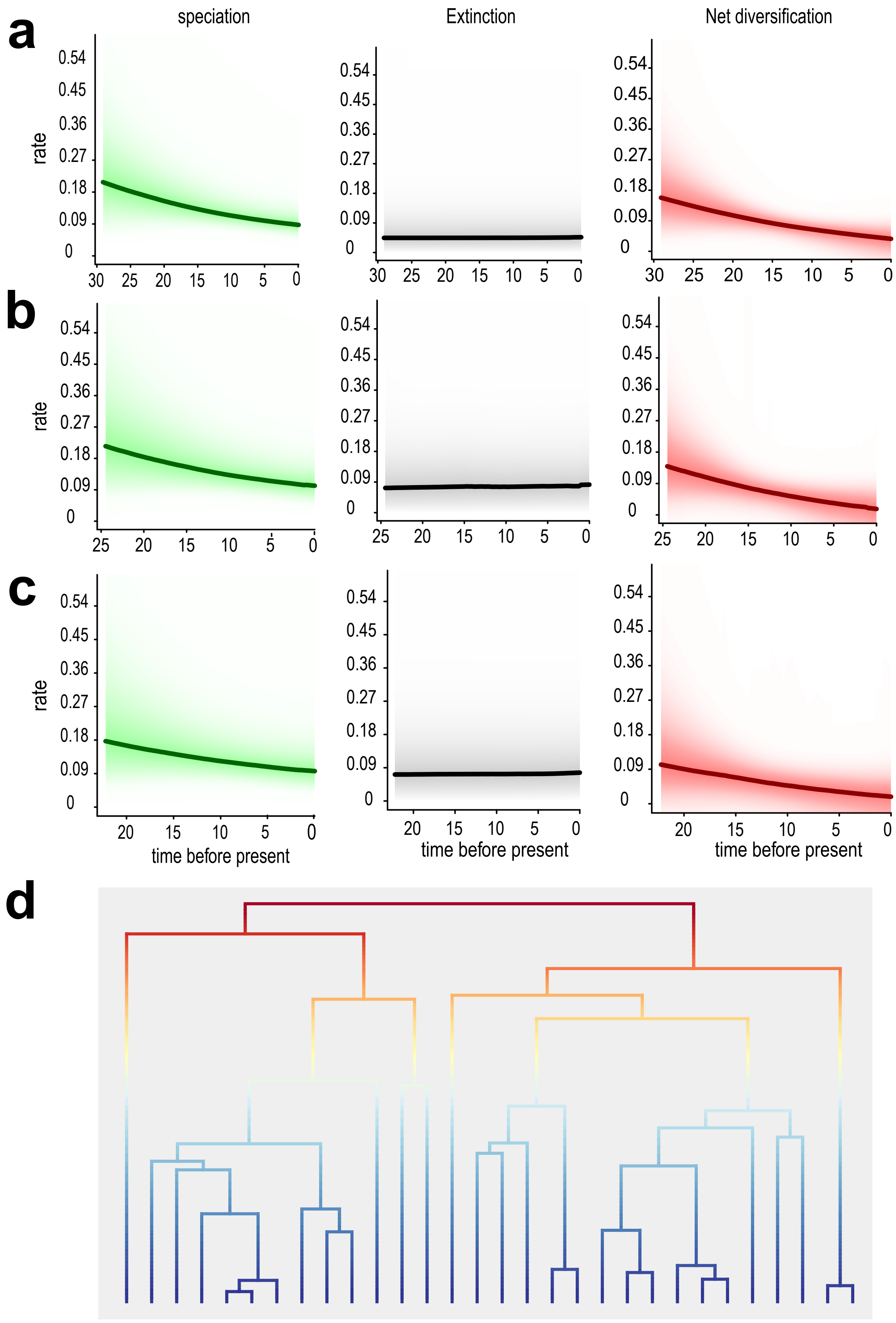
a
b
c
d
speciation
Extinction
Net diversification
rate
0.54
0.45
0.36
0.27
0.18
0.09
0
30
25
20
15
10
5
time before present

Figure S3

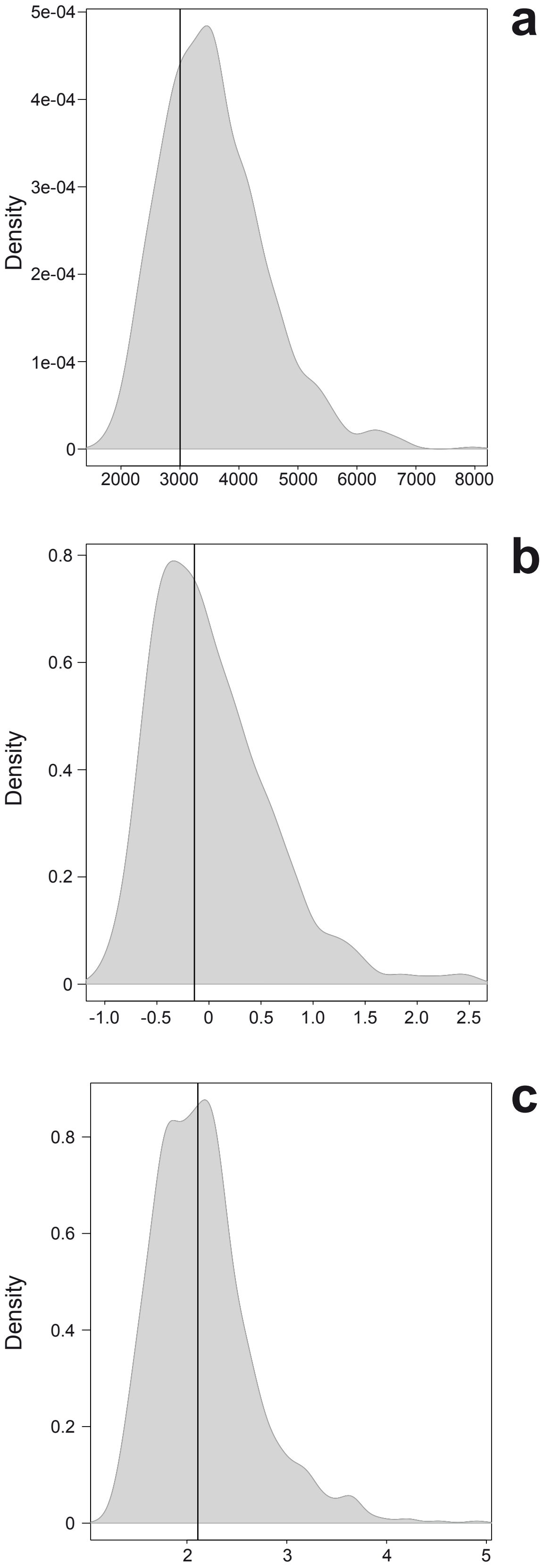

a
Density
5e-04
4e-04
3e-04
2e-04
1e-04
0
2000
3000
4000
5000
6000
7000
8000
b
Density
0.8
0.6
0.4
0.2
0
-1.0
-0.5
0
0.5
1.0
1.5
2.0
2.5
c
Density
0.8
0.6
0.4
0.2
0
2
3
4
5

Figure S4

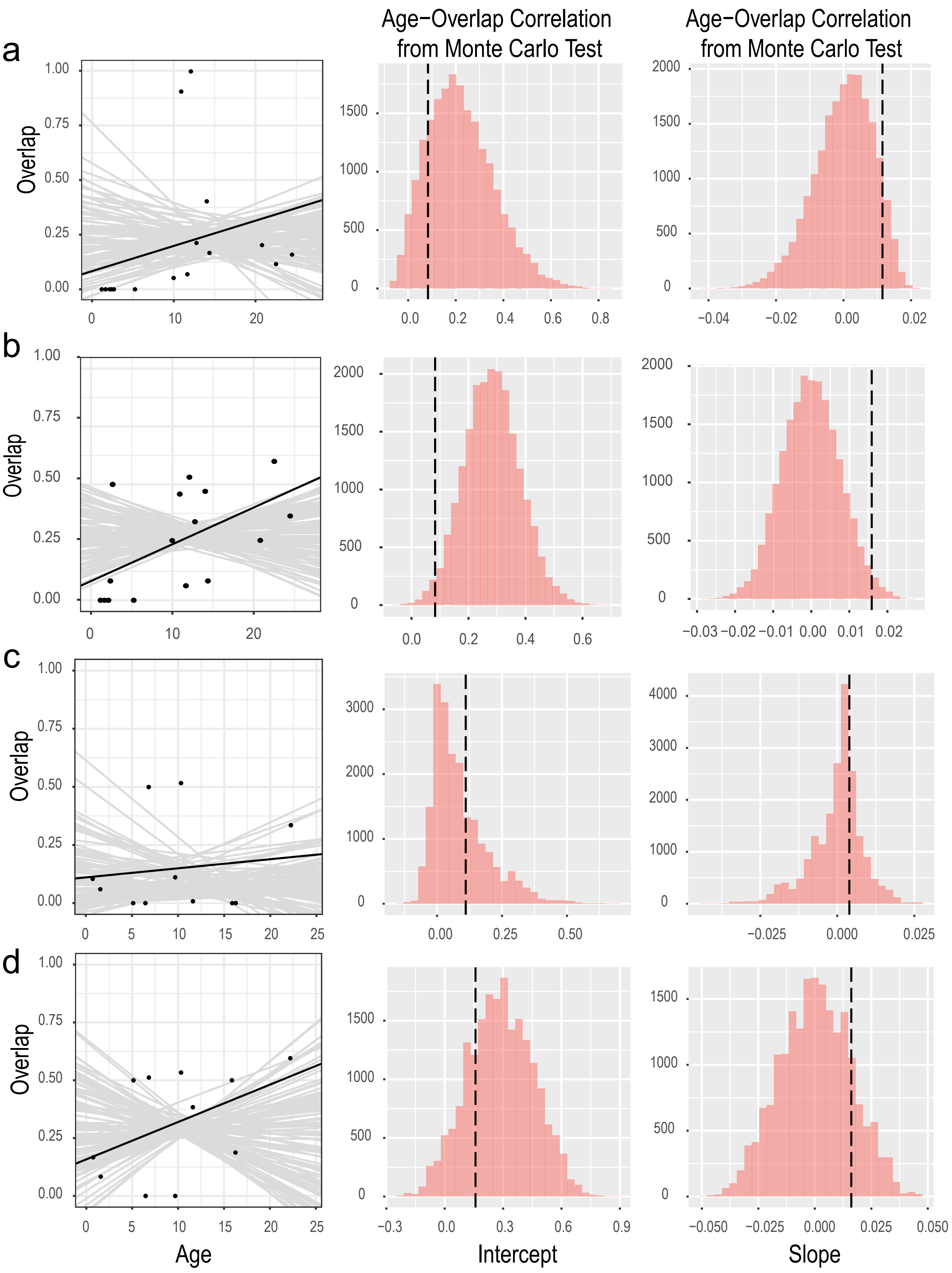
a
b
c
d
Age−Overlap Correlation from Monte Carlo Test
Age−Overlap Correlation from Monte Carlo Test
Overlap
Age
Intercept
Slope

Figure S5

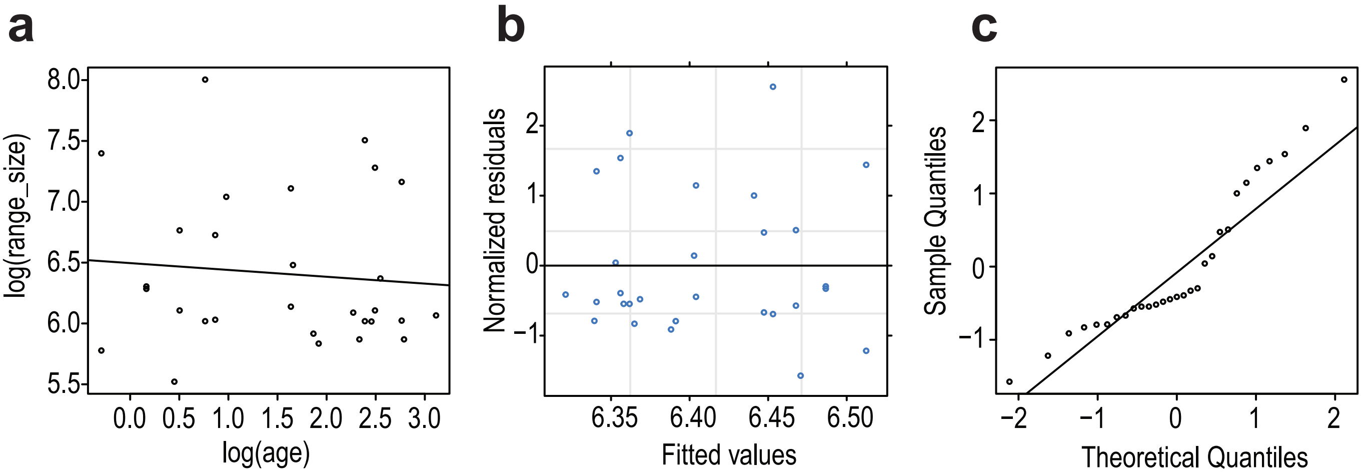
a
8.0
7.5
7.0
6.5
6.0
5.5
log(range_size)
0.0 0.5 1.0 1.5 2.0 2.5 3.0
log(age)
b
2
1
0
−1
Normalized residuals
6.35 6.40 6.45 6.50
Fitted values
c
2
1
0
−1
Sample Quantiles
−2 −1 0 1 2
Theoretical Quantiles

Figure S6

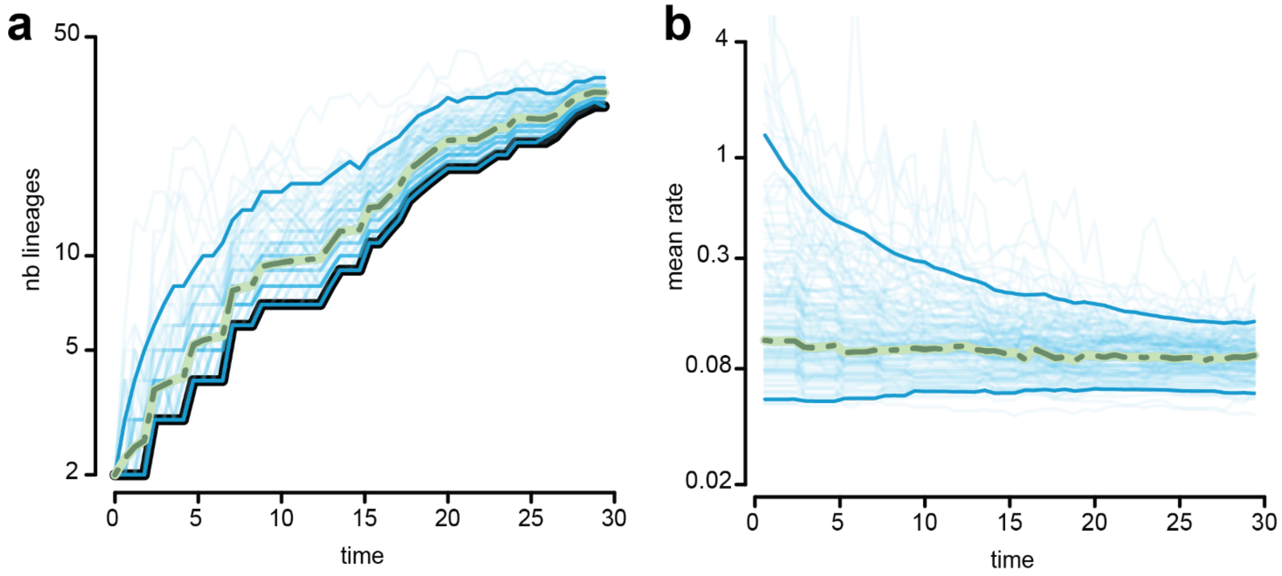

a
50
10
5
2
nb lineages
0
5
10
15
20
25
30
time
b
4
1
0.3
0.08
0.02
mean rate
0
5
10
15
20
25
30
time

Figure S7

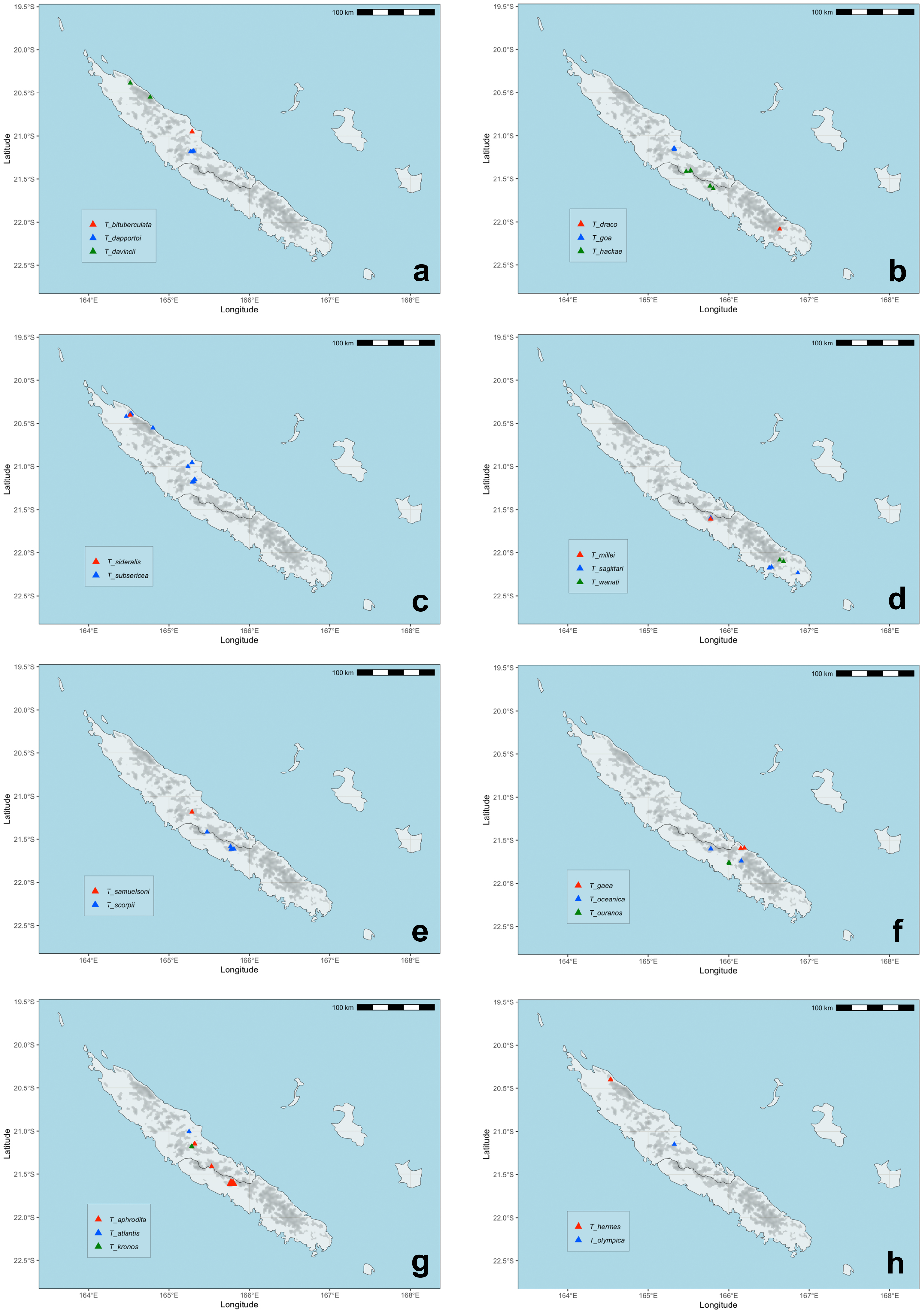
a
T_bituberculata
T_dapportoi
T_davincii
b
T_draco
T_goa
T_hackae
c
T_sideralis
T_subsericea
d
T_milleii
T_sagittari
T_wanati
e
T_samuelsoni
T_scorpii
f
T_gaea
T_oceanica
T_ouranos
g
T_aphrodita
T_atlantis
T_kronos
h
T_hermes
T_olympica
100 km
Latitude
Longitude
19.5°S
20.0°S
20.5°S
21.0°S
21.5°S
22.0°S
22.5°S
164°E
165°E
166°E
167°E
168°E
